# Molecular identification of blood meals in mosquitoes (Diptera, Culicidae) in urban and forested habitats in southern Brazil

**DOI:** 10.1371/journal.pone.0212517

**Published:** 2019-02-19

**Authors:** Camila Silva Santos, Marcio Roberto Pie, Tatiana Carneiro da Rocha, Mario Antonio Navarro-Silva

**Affiliations:** 1 Departamento de Zoologia, Laboratório de Morfologia e Fisiologia de Culicidae e Chironomidae, Universidade Federal do Paraná, Curitiba, Paraná, Brazil; 2 Departamento de Zoologia, Laboratório de Dinâmica Evolutiva e Sistemas Complexos, Universidade Federal do Paraná, Curitiba, Paraná, Brazil; 3 Departamento de Farmácia, Laboratório de Saúde Pública e Ambiental, Universidade Federal do Paraná, Curitiba, Paraná, Brazil; Centro de Pesquisas René Rachou, BRAZIL

## Abstract

The study of host associations of mosquitoes (Diptera, Culicidae) provides valuable information to assist in our understanding of a variety of related issues, from their life-history to the entomological surveillance of pathogens. In this study, we identified and characterized mosquito blood meals from both urban and forested areas in the city of Paranaguá, state of Paraná, Brazil, by analyzing the amplification of host DNA ingested by mosquitoes under different storage conditions and digestion levels. Host DNA preservation was evaluated in fresh blood meals according to storage duration (30 to 180 days) and temperature (-20°C / -80°C) and, in digested blood, according the degree of digestion classified on the Sella scale. Molecular analysis of blood meals was based on DNA extraction and amplification of a fragment of the mitochondrial COI gene. We determined that, up to180 days of storage, the evaluated temperatures did not influence the preservation of fresh blood meals DNA, whereas the amplification success was increasingly reduced over the course of the digestion process. The species *Anopheles cruzii*, *Aedes fluviatilis*, *Aedes scapularis*, *Psorophora ferox*, *Culex quinquefasciatus*, *Culex mollis*, and *Culex intrincatus*, together with specimens representing four subgenera and one genus of Culicidae [*Ae*. (*Ochlerotatus*), *Cx*. (*Culex*), *Cx*. (*Melanoconion*), *Cx*. (*Microculex*), and *Limatus*, respectively] had their blood meals identified. Their diverse host use was evidenced by the identification of 19 species of vertebrate host, namely two amphibians, three mammals and 14 birds. Birds were the most commonly identified host in blood meals. These results not only show the diversity of mosquito hosts, but also underscore the challenges involved in monitoring arboviruses of public health importance, given potential combinations of host use for each mosquito species.

## Introduction

Hematophagy is shared by females of most mosquito species [1], which use energy from blood digestion mainly for egg production and maturation, thus increasing their reproductive efficiency [2]. Blood meals might consist of blood from several host taxa, such as worms, leeches [3], fishes [4], amphibians, reptiles, birds, and mammals [5]. A species-specific host preference is an innate characteristic, with a genetic basis, but that is modulated by factors that influence patterns of search and choice of the host, such as environmental conditions and host characteristics [6].

The contact with different hosts can allow for the use of novel vertebrate species in the transmission of pathogens [7]. A less diversified host use allows for some pathogens to come into contact and to adapt to specific hosts [8,9]. On the other hand, an opportunistic zoophilic behavior can lead to the adaptation and transmission of pathogens to different species of vertebrate [10,11]. Understanding the host use patterns of mosquitoes can help in our understanding of their life-history, as well as the impact of host choice on their survival, reproduction, and in the transmission ecology of mosquito-vectored pathogens [12–15]. In addition, this knowledge is crucial for efforts related to entomological surveillance [16], providing information that can help monitoring vectors, particularly in the context of environmental disturbance [17].

Several approaches have been used to identify the blood meals of mosquitoes, such as serological and molecular methods. Assays such as precipitin tests and ELISA (Enzyme Linked Immunosorbent Assay), which were common prior to PCR-based blood meal analysis, tended to show low specificity, given that identification was based on tests that only indicated groups of vertebrates as potential hosts [18–20]. Currently, species level identification is achieved using molecular methods based on the DNA in ingested blood [21,22]. The polymerase chain reaction (PCR) followed by DNA sequencing of the PCR product is among the most direct and specific approaches, being ideal to the study of hematophagous arthropods with diverse host use, particularly in the case of wild animals [13].

Molecular methods require maximum integrity of the DNA molecule for the optimal detection and amplification of the target fragment [23]. In arthropods with blood meals, DNA degradation results mainly from the process of digestion and/or due to the storage conditions from the collection of the specimens to the DNA extraction in the laboratory [24]. The degree of blood digestion thus determines the PCR amplification and the identification of the vertebrate host [25,26]. Likewise, an inadequate preservation of the blood-engorged mosquito in terms of temperature and time since collection might reduce the chances of positive identifications [23,27].

Given that mosquitoes collected in the field show different degrees of digestion in their blood meal, it is thus important to assess the ideal storage conditions of mosquito blood meals after field collection. In addition, although storage at -80°C is the ideal way to preserve blood DNA, it is necessary to determine the temperature limits and duration of storage for mosquitoes used in studies seeking to identify their host choice [27]. Thus, the goals of the present study were: i) to analyze the impact of the degree of blood digestion, temperature, and time of storage of blood-engorged females in the DNA amplification of their vertebrate host; ii) to determine the blood sources of mosquitoes collected in urban and forested areas in a region of the Atlantic Forest in southern Brazil. The obtained results can help in the development of entomological surveillance protocols, given that the storage conditions and the process of blood digestion analyzed in the present study have been shown to be critical in the identification of blood sources. In addition, this study provides important information to our understanding of the host use patterns of mosquitoes in natural and urban environments.

## Material and methods

### Ethics statements

The protocol for the use of animals in the laboratory was approved by the Commission on Ethics for the Use of Animals of the Institute of Biological Sciences of the Universidade Federal do Paraná (CEUA/BIO-URPF) (Protocol number 719). All procedures for manipulation and animal care were carried out in strict conformity with the recommendations by the training manual of that commission. The collection of mosquito specimens in the field was carried out in a private urban area, which was authorized by the respective owners. In the forested area, in a public property, the collection permit was issued by the Instituto Ambiental do Paraná. In both areas, the collection of specimens did not involve threatened or protected species and did not entail losses for any vertebrate animal, nor risks to human health.

### Mosquitoes

In order to assess the effects of temperature and storage duration on the amplification of blood DNA, we used females of *Aedes aegypti* belonging to a Rockefeller strain. These females were approximately 30 days old and were reared and kept in the Laboratório de Morfologia e Fisiologia de Culicidae e Chironomidae of the Universidade Federal do Paraná under temperatures between 25–29° C, 70–80% relative humidity, and 12:12 h (light:dark) photoperiod. Daily, these mosquito females had a 10% sucrose solution available for feeding. For the blood meal, anesthetized mice (*Mus musculus*) became available for approximately 40 minutes, following a protocol approved by the committee on ethics for animal experimentation. The assessment of the effect of the degree of blood digestion on DNA amplification, as well as the identification of vertebrate hosts, involved females collected in the field.

### Study area

**Fig 1:**
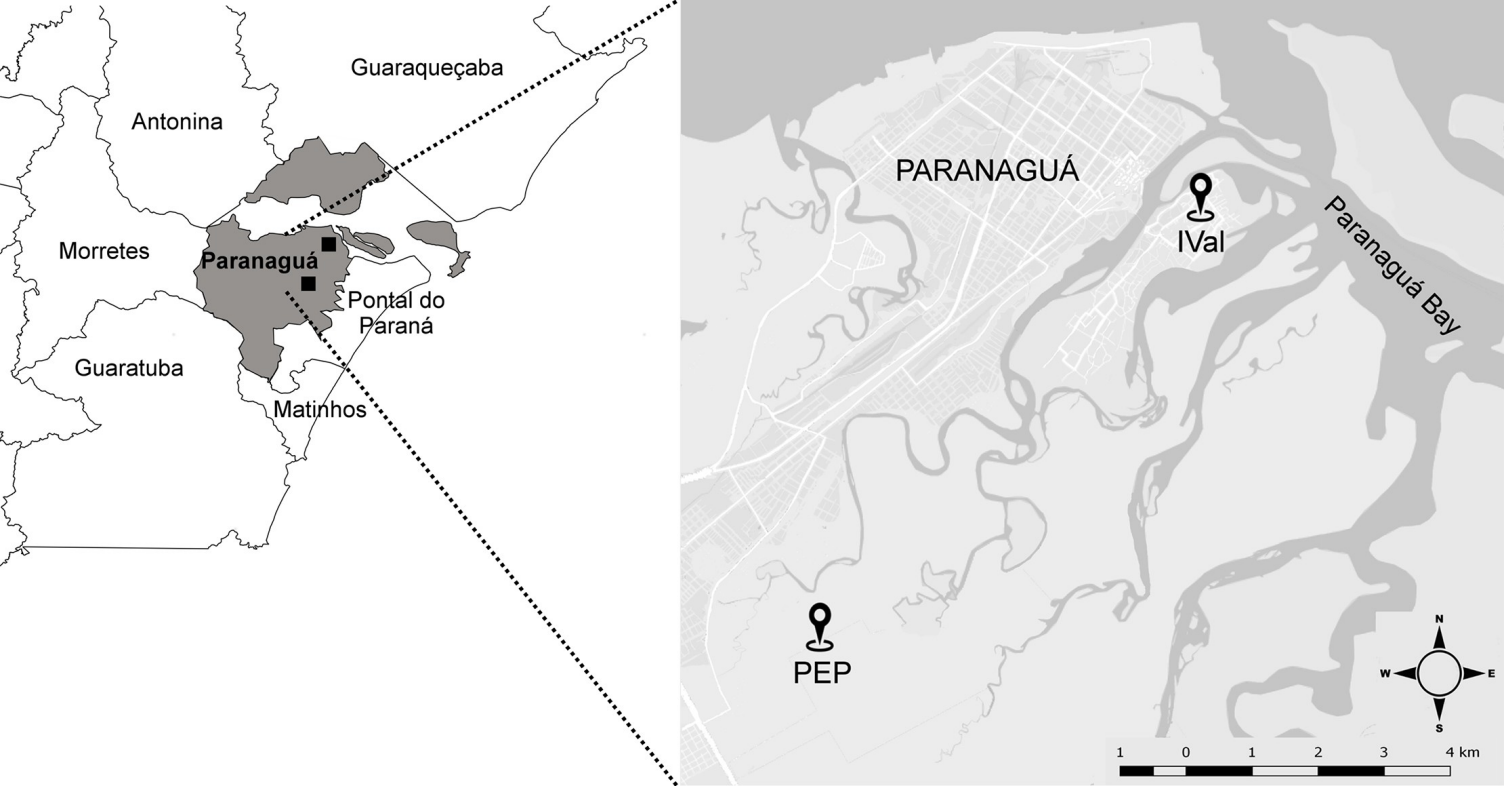
Municipality of Paranaguá, located in the coastal region of the state of Paraná, Brazil. Location of the urban site (IVal) and the forested site (PEP).

The municipality of Paranaguá, Paraná, Brazil (25°31’14” S, 48°30’34” W), located in the coastal region of the state, has an annual rainfall between 2,000 and 2,200 mm, with well-distributed rain throughout the year and no clearly defined dry season. There is predominance of humid subtropical climate (Cfa), based on the classification of Koeppen [28]. Two areas were studied in this municipality: an urban site (Ilha dos Valadares–IVal) and a forested site (Parque Estadual do Palmito–PEP) (Fig 1). The IVal has an area of about 5 km^2^ and a population of approximately 26,000 inhabitants (in 2010) [29]. The PEP is a remnant of Atlantic Forest with an area of 17.824 km^2^, being composed of lowland dense ombrophilous forests, mangroves, and coastal shrublands. Given that it is a permanently protected area, the park allows for public visitation and scientific research. Studies in the park have indicated the presence of anurans [30], birds [31], bats [32], and a primate [33] (Table 1).

**Table 1:** Species of vertebrate detected in the PEP according to bibliographical data and recorded between 2006 and 2014.

Vertebrate	Reference
**Aves**	
*Phalacrocorax brasilianus; Ardea cocoi; Casmerodius albus; Egretta thula; Egretta caerulea; Butorides striatus; Nycticorax nycticorax; Nyctanassa violacea; Platalea ajaja; Coragyps atratus; Cairina moschata; Micrastur ruficollis; Milvago chimachima; Polyborus plancus; Aramides cajanea; Vanellus chilensis; Charadrius semipalmatus; Actitis macularia; Columba cayennensis; Amazona braziliensis; Tyto Alba; Otus choliba; Asio stygius; Nyctibius griseus; Chordeiles acutipennis; Nyctidromus albicollis; Aphantochroa cirrhochloris; Ceryle torquata; Chloroceryle amazona; Chloroceryle americana; Chloroceryle aenea; Picumnus cirratus; Veniliornis spilogaster; Thamnophilus caerulescens; Camptostoma obsoletum; Attila rufus; Myiarchus ferox; Pitangus sulphuratus; Legatus leucophaius; Tyrannus melancholicus; Progne chalybea; Notiochelidon cyanoleuca; Stelgidopteryx ruficollis; Cyanocorax caeruleus; Troglodytes musculus; Turdus rufiventris; Turdus amaurochalinus; Vireo chivi; Parula pitiayumi; Geothlypis aequinoctalis; Ramphocelus bresilius; Cacicus haemorrhous; Cacicus chrysopterus*.	[34]
**Mammalia**	
Bats—*Anoura caudifer; Artibeus fimbriatus; Artibeus lituratus; Artibeus obscurus; Artibeus planirostris; Carollia perspicillata; Chiroderma doriae; Desmodus rotundus; Glossophaga soricina; Micronycteris megalotis; Pygoderma bilabiatum; Sturnira lilium; Sturnira tildae; Vampyressa pusilla; Molossus rufus; Eptesicus diminutus; Eptesicus taddeii; Myotis nigricans; Myotis riparius*.	[32]
Primates—*Callithrix penicillata*	[33]

### Sampling, storage, and identification of mosquito females

In the laboratory, 36 blood-engorged *Ae*. *aegypti* females (Rockefeller strain) were killed by freezing at -20°C immediately after full blood engorgement, and were individually placed in 1.5 mL microtubes. Half of those specimens were stored at -20°C, whereas the other half was kept at -80°C. Every 30 days, over a period of 180 days, three stored samples at each temperature were processed to identify their blood meal.

In the field, resting mosquitoes were collected between October of 2016 and March of 2017, in eight days of sampling, average of one collection per month, except January (no collections), February (three), and March (two), alternating between the morning (between 8:00 AM and 12:00 AM) and the afternoon (between 1:00PM and 4:00PM). Mosquitoes were collected with the simultaneous use of two or three Nasci aspirators attached to a 12V battery. The mosquito receptacle was changed every 10 min, for a total sampling effort of 710 min (130 min in IVal and 580 min in PEP). In IVal, sampling was carried out both within and around housing areas, where as in PEP sampling was conducted in transect across the main trail by walking perpendicularly to the edge of the forest. Three points were sampled across a total travel distance of 1.2 km.

**Fig 2:**
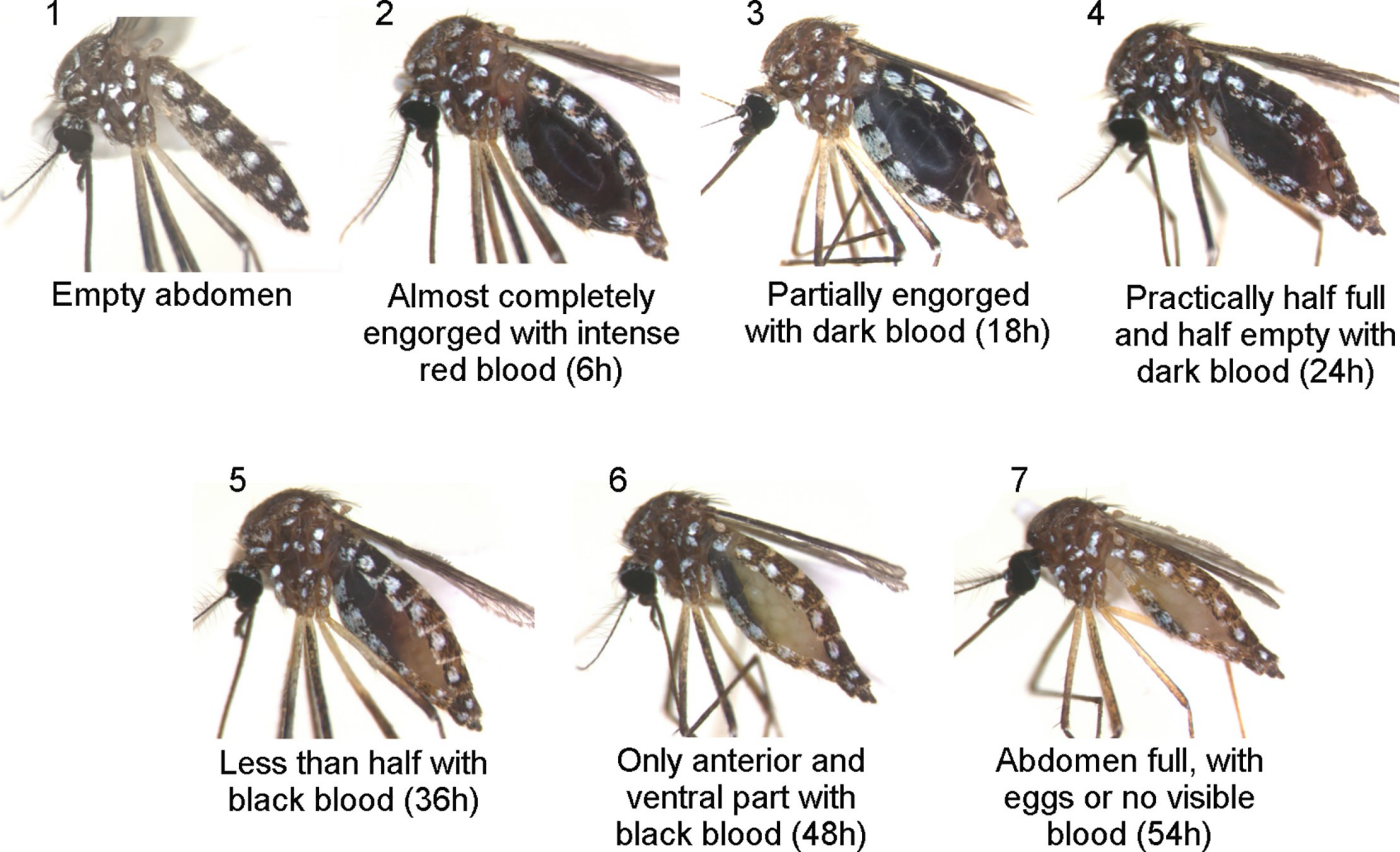
Females of *Aedes aegypti* at different degrees of digestion, based on the Sella scale. Each specimen is indicated with the corresponding approximate digestion time (in h).

Field-collected mosquitoes were killed by freezing in a cooler with liquid nitrogen, in which they were also transported under a temperature that did not exceed -4°C. In the laboratory, mosquitoes were stored at -80°C until specimens were identified. Using a refrigerated surface and a stereoscopic microscope, engorged females were identified at the species level using dichotomous keys [35–38]. The degree of digestion in the engorged blood was classified according to the Sella scale, following Detinova et al. [39] (Fig 2). Females classified between 2 and 6 were housed in individual microtubes and stored at -80°C until the molecular analysis of their blood meals.

### Identification of the blood meals

DNA was isolated from individual engorged females using the HotShot protocol developed by Truett et al. [40]. Using tweezers and sterile pipette tips, the abdomen of each analyzed female was removed and placed whole within 0.2 mL tubes containing 50 μL of lysis buffer (NaOH 25mM + EDTA 0.2 mM, pH 12.0). The blood was mixed with the solution and the visible parts of the abdomen were then removed. Samples were incubated in a thermocycler at 95°C for 30 min and later placed in an ice bath for four to five min. Fifty μL of neutral buffer (Tris-HCl 40 mM, pH 5.0) were then added and thoroughly homogenized the samples. Samples were kept at -20°C until being amplified by PCR.

The template DNA was amplified using a nested PCR protocol using universal vertebrate primers (M13BC-FW and BCV-RV1; M13 and BCV-RV2) (Table 2) developed by Alcaide et al. [41] that has as its target a ~800-bp fragment of the cytochrome c oxidase I gene (COI). A slightly modified protocol for the first PCR was done with 30 μL final volume containing 1X of enzyme buffer (Sigma), 3.16 mM of MgCl_2_ (Thermo Scientific), 0.5 mM of dNTP mix (Amresco), 10 μg of BSA (Bovine Serum Albumin), 5% of DMSO, 0.66 μM of each of the primers (M13BC-FW and BCV-RV1), 1U of Taq DNA Polymerase (Sigma), and a concentration of extracted DNA between 70 and 150 ng/μL. Thermocycling conditions included an initial denaturation phase for 4 min at 94°C, followed by 39 cycles of 40 s at 94°C, 40 s at 45°C and 1 min at 72°C, with a final extension of 7 min at 72°C, in a Bio-Rad thermocycler. The second PCR was carried out in a final volume of 30 μL including 1X of enzyme buffer (Sigma), 5.66 mM of MgCl_2_ (Thermo Scientific), 0.66 mM of dNTP mix (Amresco), 5 μg of BSA (Bovine Serum Albumin), 5% of DMSO, 0.33 μM of each primer (M13 and BCV-RV2), 1U of Taq DNA Polymerase (Sigma), and 1.0 μL of PCR product of the first reaction. Thermocycling conditions included an initial denaturation of 3 min at 94°C, followed by a touchdown protocol of 16 cycles of reduction in annealing temperature from 60°C to 45°C (-1°C per cycle) with 1 min of extension at 72°C and 40 s of denaturation at 94°C, followed by 24 cycles of 40 s at 94°C, 45°C and 72°C, with a final extension of 7 min at 72°C. Each reaction included a negative control of autoclaved Milli-Q water instead of template DNA, as well as a positive control established in preliminary tests using Rockefeller strain *Ae*. *aegypti* females that ingested only mice blood and went through the whole process of identifying the blood meal, including sequencing.

**Table 2:** Primers used for the amplification of DNA fragments of the COI gene for vertebrates and mosquitoes.

Primer	Sequence (5’–> 3’)	Target gene (identified taxon)
M13BC-FW	TGT AAA ACG ACG GCC AGT HAA YCA YAA RGA YAT YGG NAC	COI (Vertebrate)
BCV-RV1	GCY CAN AYY ATN CYY RTR W	COI (Vertebrate)
M13	GTA AAA CGA CGG CCA GTG	COI (Vertebrate)
BCV-RV2	ACY ATN CCY ATR TAN CCR AAN GG	COI (Vertebrate)
LCO 1490	GGT CAA CAA ATC ATA AAG ATA TTG	COI (Culicidae)
HCO 2198	TAA ACT TCA GGG TGA CCA AAA AAT CA	COI (Culicidae)

The products of the second PCR were subjected to electrophoresis in a 1% agarose gel. Amplification success was used to assess the impact of storage conditions and degree of digestion on amplification success. To determine the blood meals, field samples that had been successfully amplified were purified using a QIAquick PCR Purification Kit (Qiagen) according to the manufacturer’s instructions. Purified DNA was subject to Sanger sequencing reactions using BigDye Terminator v3.1 Cycle Sequencing Kit (Applied Biosystems) and sequenced on an ABI 3730 DNA Analyser (Applied Biosystems) at the Centro de Pesquisas sobre o Genoma Humano e Células-Tronco of the Universidade de São Paulo.

### Molecular identification of Culicidae

Given the damage caused by the collection through suction method to some important structures (e.g. scales and legs), molecular identification was also used to determine the species, subspecies, or genera of collected mosquitoes. Only females with uncertain morphological identification and that had their blood meal successfully determined were included in this analysis. All structures present after separating the abdomen (i.e. head, thorax, and legs) were used for DNA extraction, following the protocol developed by Bona et al. [42]. The amplification of a 650-bp fragment of the COI gene was carried out using the universal invertebrate primers LCO 1490 and HCO 2198 [43] (Table 2). The used PCR protocol was based on Wang et al. [44] as follows. PCR included 1X enzyme buffer (Sigma), 3.16 mM of MgCl_2_ (Thermo Scientific), 0.2 mM of dNTP mix (Amresco), 0.4 μM of each of the primers, 1U of Taq DNA Polymerase (Sigma), and a volume of DNA extract with a concentration of approximately 40 ng/μL to reach a final volume of 30 μL. Thermocycling conditions included an initial denaturation for 1 min at 94°C, followed by six cycles of 40 s at 94°C, 40 s at 45°C and 1 min at 72°C, 36 cycles of 40 s at 94°C, 40 s at 51°C and 1 min at 72°C, with a final extension of 5 min at 72°C. The verification of amplification, purification, and, sequencing were the same as those used to identify blood meals, as well as the analysis of the resulting sequences.

### Sequence analysis

The obtained sequences were edited using BioEdit Sequence Alignment Editor v. 7.2.5 [45,46] and aligned using MUSCLE, as implemented in MEGA 6 [47]. Sequence identification was conducted by comparison with those deposited in GenBank (NCBI: National Center for Biotechnology Information– https://blast.ncbi.nlm.nih.gov/Blast.cgi) and BOLD (Barcode of Life Data System -http://www.barcodinglife.org/index.php/IDS_OpenIdEngine). A vertebrate host was considered as identified when sequence similarity was ≥ 98%. As some species of Culicidae that were present in the PEP [48] did not contain genetic data in GenBank or BOLD, by standardization, we considered as identified species only those with similarity equal to 100%. Similarly values below those cut-off values were considered as identification to the genus or subgenus levels.

### Data analysis

Amplification success at each temperature was assessed according to the total number of successfully amplified samples at each storage duration. For mosquitoes collected in the field, scores based on the digestion scale were related to the number of successful amplifications using a Wilcoxon Rank Sum Test, carried out in R version 3.5.0 [49].

## Results

**Fig 3:**
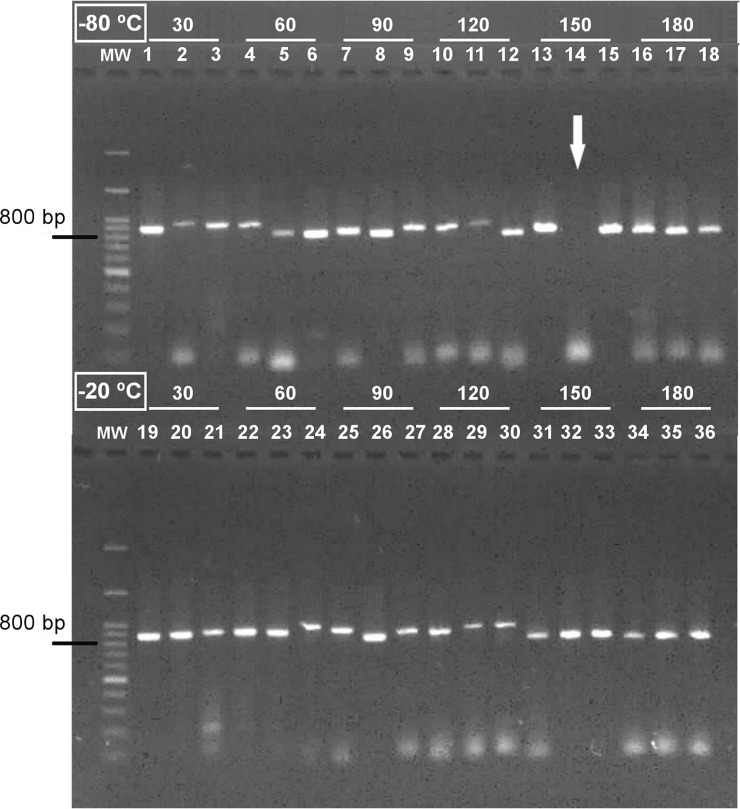
Amplification of DNA present in completely engorged *Aedes aegypti* females that were stored under -80°C or -20°C for 30 to 180 days until DNA extraction. Wells 1–18: females stored at -80°C, with a 94,4% of amplification success. Arrow indicates sample 14, stored for 150 days that did not lead to amplification. Wells 19–36: females stored at -20°C with 100% amplification success. MW: 100-bp molecular weight ladder.

Samples that remained stored for 30 to 180 days at -20°C resulted in 100% amplification of host DNA from completely engorged *Ae*. *aegypti* females. Similar results were obtained for samples kept at -80°C, except that one sample did not result in positive amplification, leading the amplification success under those conditions to be slightly lower (i.e. 94.4%, Fig 3).

Of all 3.087 field-collected mosquitoes, 1.951 were female and, of those, 155 (7.9%) were blood-engorged. Of those females, 148 allowed for the visualization of the degree of blood meal digestion and were classified according to the Sella scale (between 2 and 6). Based on that set of females, 52 (35.1%) led to positive DNA amplification, with the highest success rates in females classified as either 2 and 3. The degree of blood digestion was inversely related to amplification success (Wilcoxon rank sum test, W = 3274.5, p = 0.0012), with a particular reduction in the beginning of class 4 females (Table 3).

**Table 3:** Total of engorged females collected in IVal and PEP classified according to the Sella scale. Amount and percentage of amplification per scale.

Sella scale	Engorged females	Successful amplifications (%)
**2**	50	24 (48)
**3**	30	14 (47)
**4**	12	3 (25)
**5**	17	4 (24)
**6**	39	7 (18)
**Total**	148	52

Of the 155 engorged females collected in IVal and PEP, 54 (34.8%) had their host identified through blood meal analysis. As the molecular identification of mosquitoes has been shown to be efficient, mainly of cryptic species [44], through this method and the use of morphological characters, we identified seven mosquito species (*An*. *cruzii*, *Ae*. *fluviatilis*, *Ae*. *scapularis*, *Ps*. *ferox*, *Cx*. *quinquefasciatus*, *Cx*. *mollis*, and *Cx*. *intrincatus*) and specimens that represented four subgenera and one genus of Culicidae [*Ae*. (*Ochlerotatus*), *Cx*. (*Culex*), *Cx*. (*Melanoconion*), *Cx*. (*Microculex*), and *Limatus*, respectively], all analyzed with respect to their blood source (Table 4). These numbers did not include females whose blood meals were successfully amplified, but that did not show similarity with vertebrate species deposited in GenBank or BOLD (Table 4). These sequences showed a high number of double peaks in the electropherograms, which suggested that their blood meals included more than one host species in the same gonotrophic cycle. Of the 54 studied females, only seven mosquito specimens were identified through molecular techniques (Table 4).

**Table 4:** Mosquito species for which blood-engorged females were collected in IVal and PEP, average Sella score of total engorged females and number of blood meals identified by species. Specimens with molecular identification are shown with corresponding GenBank accession numbers.

Mosquito species	Engorged females	Average Sella score	Identified blood meals (%)
**Subfamily Anophelinae**			
*Anopheles* (*Kerteszia*) *cruzii* Dyar & Knab, 1908	2	2	2 (3.70)
**Subfamily Culicinae**			
**Tribe Aedini**			
*Aedes* (*Stegomyia*) *aegypti* (Linnaeus, 1762)	1	3	-
*Aedes* (*Georgecraigius*) *fluviatilis* (Lutz, 1904)	3	5 ± 1.73	1 (1.85)
*Aedes* (*Ochlerotatus*) *scapularis* (Rondani, 1848)	10	3.4 ± 1.58	3 (5.55)
*Aedes (Ochlerotatus)* spp^a^(MH879308; MH879310)	2	4.5 ± 2.12	2 (3.70)
*Aedes* spp.	5	4.25 ± 1.5	-
*Psorophora* (*Janthinosoma*) *ferox* (von Humboldt, 1819)	5	4.67 ± 2.31	2 (3.70)
*Psorophora* (*Janthinosoma*) sp.	2	4.5 ± 2.12	-
**Tribe Culicini**			
*Culex* (Culex) *quinquefasciatus* Say, 1823	92^c^	3.57 ± 1.59	36 (66.70)
*Culex (Culex) mollis* Dyar & Knab, 1906^b^(MH879306)	1	6	1 (1.85)
*Culex (Culex)* sp.^a^(MH879309)	1	2	1 (1.85)
*Culex (Melanoconion) intrincatus* Brèthes, 1916 ^b^ (MH879304)	1	2	1(1.85)
*Culex (Melanoconion)* sp.^a^(MH879305)	1	2	1 (1.85)
*Culex* (*Microculex*)spp.	14	4.36 ± 1.55	3 (5.55)
*Culex* spp.	10	3.89 ± 1.83	-
**Tribe Sabethini**			
*Limatus* sp.	2	6	1 (1.85)
*Sabethes* spp.	2	6	-
*Wyeomyia* sp.^a^(MH879307)	1^c^	3	-
**Total**	155	3.76 ± 1.64	54 (100)

^a^ Molecular identification (identity<100%)

^b^ Molecular identification (identity = 100%)

^c^ One specimen was sequenced but not identified given that there were more than one host species in the blood meal.

In IVal, we were able to identify the origin of the blood meal of 29 mosquito females of the following species *Cx*. *quinquefasciatus* (n = 27), *Cx*. *intrincatus* (n = 1), *Ae*. *scapularis* (n = 1), which fed on avian (68.96%) and mammal blood (31.04%). Most blood meals of *Cx*. *quinquefasciatus* were identified as originating from chicken (*Gallus gallus*), followed by dogs (*Canis lupus familiaris*), humans (*Homo sapiens*), and passerine birds (*Passer domesticus*, and *Turdus amaurochalinus*). The blood meal of *Cx*. *intrincatus* led to the identification of the heron *Nyctanassa violacea*, whereas the blood meal of *Ae*. *scapularis* was identified as coming from a domestic dog (Table 5). All of the blood meals identified as human blood were detected in females collected within a human residence, as well as a female that fed on the blood of a chicken. The remaining vertebrates were identified from the blood meals of females collected in the vicinity of human housing.

**Table 5:** Blood meals of mosquito females collected in IVal in October and November of 2016 using Nasci aspirators. Accession numbers indicate sequences deposited in GenBank corresponding to the identified vertebrate species.

Mosquito species	Specimens	Aves	Mammalia
*Aedes scapularis*	1	-	*Canis lupus familiaris* (1) (MH814481)
*Culex intrincatus*	1	*Nyctanassa violacea* (1) (MH791070)	-
*Culex quinquefasciatus*	27	*Gallus gallus* (17) (MH814458 to MH814474)	*Canis lupus familiaris* (4) (MH814477 to MH814480)
		*Passer domesticus* (1) (MH791071)	*Homo sapiens* (4) (MH791060 to MH791063)
		*Turdus amaurochalinus* (1) (MH791078)	
**Total**	29	20	9

In PEP, 25 females had their blood meal identified and included the following species: *An*. *cruzii* (n = 2), *Ae*. *fluviatilis* (n = 1), *Ae*. *scapularis* (n = 2), *Ps*. *ferox* (n = 2), *Cx*. *quinquefasciatus* (n = 9), *Cx*. *mollis* (n = 1), *Aedes* (*Ochlerotatus*) sp. (n = 2), *Culex* (*Culex*) sp. (n = 1), *Culex* (*Melanoconion*) sp. (n = 1), *Culex* (*Microculex*) sp. (n = 3), and *Limatus* sp. (n = 1). Fourteen species of vertebrates were used as host, including wild birds (n = 17, 68%), mammals (n = 6, 24%), and amphibians (n = 2, 8%). The genus *Culex* only had wild birds as their blood source, except for the subgenus *Microculex* which included a bird and two amphibians (*Scinax argyreornatus* and possibly *Trachycephalus* sp., both hylids). The blood meals of *Ae*. *scapularis* were identified as blood from birds and horse, and another specimen of the subgenus *Ochlerotatus* and a female of *Limatus* fed on avian blood. All blood meals of *An*. *cruzii*, *Ae*. *fluviatilis*, and *Ps*. *ferox* were identified as human blood (Table 6).

**Table 6:** Blood meals of mosquito species collected in PEP using aspirators between December of 2016 and March of 2017. Accession numbers indicate sequences deposited in GenBank corresponding to the identified vertebrate species.

Mosquito species	Specimens	Amphibia	Aves	Mammalia
*Anopheles cruzii*	2			*Homo sapiens* (2) (MH791067; MH791068)
*Aedes fluviatilis*	1			*Homo sapiens* (1) (MH791064)
*Aedes scapularis*	2		*Pyriglena leucoptera* (1) (MH791073)	*Equus caballus* (1) (MH814457)
*Aedes* (*Ochlerotatus*) spp.	2		*Crypturellus* spp.(2)^a^ (MH898861; MH898862)	
*Psorophora ferox*	2			*Homo sapiens* (2) (MH791065; MH791066)
*Culex quinquefasciatus*	9		*Conopophaga melanops* (1)(MH814455)	
			*Herpsilochmus rufimarginatus* (3) (MH814475; MH814476; MH791059)	
			*Malacoptila striata*(1) (MH791069)	
			*Tinamus solitarius*(1)^b^ (MH879303)	
			*Turdus albicollis*(1) (MH791075)	
			*Turdus flavipes* (1) (MH791079)	
			*Patagioenas picazuro*(1) (MH791072)	
*Culex mollis*	1		*Turdus albicollis* (1) (MH791077)	
*Culex*(*Culex*) sp.	1		*Turdus albicollis*(1) (MH791076)	
*Culex* (*Melanoconion*) sp.	1		*Conopophaga melanops* (1)(MH814456)	
*Culex* (*Microculex*) spp.	3	*Scinax argyreornatus* (1) (MH791074)	*Turdus rufiventris* (1) (MH791080)	
		*Trachycephalus* sp.(1)^a^ (MH898863)		
*Limatus* sp.	1		*Crypturellus* spp.(1)^a^ (MH898860)	
**Total**	25	2	17	6

^a^ Identified vertebrate with identity< 98%

^b^ Identified vertebrate only in BOLD

## Discussion

In the case of fresh blood meals, the short-term temperature storage conditions of engorged mosquitoes (up to 180 days) had a minor impact on the amplification of host DNA. However, the degree of digestion had a strong negative influence on the amplification and identification of host DNA. Over the past few decades, it has been shown that DNA detection and amplification by PCR are more efficient for engorged mosquitoes kept at temperatures equal or below -70°C [50]. In the present study, the effects of temperature and time of storage were nearly equivalent between -20°C and -80°C to *Ae*. *aegypti* females engorged with fresh blood. Previous studies using blood samples kept at -20°C for 95 days also maintained DNA integrity for molecular analyses [51]. On the other hand, in samples stored for over 20 years, the amount and quality of preserved DNA at -20°C were reduced in relation to preservation at -80°C, such that it is advised to keep samples under ultra-low temperatures for long-term storage [52].

For completely engorged mosquitoes with little or no interference in their digestion, especially engorged with fresh blood, the storage at -20°C for up to 180 days was sufficient to achieve good results in terms of PCR amplification. Tests with only fresh blood meals have limited the establishment of temperatures and storage periods more appropriate for blood already influenced by the digestion process, which can reduce the breadth of these factors. For field-collected specimens, DNA degradation due to the digestion process is unavoidable and, together with temperature, might reduce the viability and positive amplification of blood meals [27]. However, the physical conditions for the storage of mosquitoes at 4°C or -20°C, for up to two days, did not significantly affect DNA amplification, which is mostly influenced by the degree of digestion [24].

In the present study, amplification success decreased according to the degree of the digestion. Laboratory experiments that assessed blood digestion over the course of hours [24,26,27] and field studies that measured the degree of digestion using the Sella scale [53–55] also showed this relationship. In addition, these studies were corroborated by the results of our study, which indicated that scores 2 and 3 were the most favorable for DNA amplification. In those studies, a significant reduction in amplification was detected starting on scores 5 and 6, yet this progressive reduction was detected in our study starting on score 4, which led to only 25% amplification success (Table 3).The loss of quality and quantity of DNA caused by the digestion of blood associated with other factors (e.g. extraction protocol [54], blood PCR inhibitors such as heme [56], and primer specificity and size of target fragment [57]) may have significantly affected the identification efficiency of the blood meals. The use of a universal primer whose target is a relatively long fragment, as used in the present study (about 800 bp), may also decrease the success of amplification, especially in DNA already partially degraded by digestion [57]. This factor may explain the different success rates found in blood meal amplifications of laboratory tested samples (without interference from digestion) and field samples (always observed digestion).

Despite the relative efficacy for identifying mosquito species [44], molecular identification based on DNA barcodes has already proved ineffective for some closely related species. In Brazil, some fragments of COI used in the identification of mosquito species were not sufficient to distinguish some species such as those of the subgenus *Culex* [58]. In addition, we also found that some species already recorded in our study area still do not present genetic information in the available databases (e.g. *Culex ribeirensis*). Thus, even using this molecular tool to identify mosquitoes, some specimens could not be identified at a specific level, and, in the urban and forested area, seven mosquito species (*An*. *cruzii*, *Ae*. *fluviatilis*, *Ae*. *scapularis*, *Ps*. *ferox*, *Cx*. *quinquefasciatus*, *Cx*. *mollis*, *Cx*. *intrincatus*) and specimens representing four subgenera and one genus of Culicidae [*Ae*. (*Ochlerotatus*), *Cx*. (*Culex*), *Cx*. (*Melanoconion*), *Cx*. (*Microculex*), and *Limatus*] were analyzed for the identified blood source. This analysis resulted in the identification of 19 vertebrate species as hosts (two amphibians, three mammals, and 14 birds). Given that the use of broad-spectrum molecular markers may not be sufficient to identify some of the species present in areas of high fauna richness such as PEP, success rates and specificity of identifications may be limited. However, the resulting identifications in this study allowed us to understand the host use of Culicidae species studied here and, through the identification of large groups of vertebrates, to infer about the potential risks of transmission of pathogens.

Only two species of mosquitoes (*Cx*. *quinquefasciatus* and *Ae*. *scapularis*) and one of vertebrate (*Homo sapiens*) were commonly recorded in both areas. As expected, the diversity of mosquitoes and hosts identified was higher in the forest area than in the urban area, since the PEP certainly has higher species richness [30–34]. Birds represented most of the positive identifications and this may be related to the great abundance and availability of this class in both environments studied. In IVal, during collections, it was common to observe domestic poultry breeding outside some residences and, in the PEP, wild birds contribute significantly to the composition of the registered fauna [31,34].

*Culex quinquefasciatus*, in addition to being commonly studied in both areas, was the most abundant both in terms of engorged females and in the number of identified blood meals (Table 4). In many studies of mosquito host use, this species is commonly detected in urban and rural areas, as well as in parks and forests of the Neotropics [59–62]. The ornithophilic habit was confirmed, with birds accounting for 77.8% of the identified blood meals, whereas mammals were detected in the remaining cases (only in females collected in urban areas). Given that it is considered as an opportunistic species [61], host selection between these two vertebrate classes has already been observed [60,63], which underscores the influence of the environment on the selection of blood meal sources [59]. Among mammals, the equal proportion of canine and human hosts (Table 5) did not evidence a clear preference in the selection within that class of vertebrate [64]. Chickens and passerines were the most commonly identified hosts (Tables 5 and 6), being widely used as blood sources in urban areas [65]. Both in this habitat and in the forested area, other orders of wild birds were also detected, given that the availability of other birds in that area might have facilitated their use as hosts.

The use of birds was also recorded in other specimens of *Culex*. Species of the subgenus *Culex*, such as *Cx*. *mollis*, are considered as primarily ornithophilic [5], with different species showing variation in their host use [60]. On the other hand, although some species of *Melanoconion* are primarily specialists [16], others, such as *Cx*. *intrincatus*, are considered as generalists, selecting birds, mammals, amphibians, and reptiles [5,66]. In general, and including the results on *Cx*. *quinquefasciatus*, the ornithophilic habit was particularly common in females of *Culex* (Tables 5 and 6).

The identification of blood meals in *Microculex* has also contributed to the frequency of avian blood means in our study, yet the record of amphibian hosts corroborate their tendency to select ectothermic hosts as well [67]. Given that these are mosquitoes with no known importance in terms of public health, few studies investigated and recorded their host use patterns [19,67,68]. As such, factors that influence their host preferences are still unknown.

*Aedes scapulari*s considered as a generalist in its choice of blood sources, which could be influenced by the availability and abundance of vertebrate hosts [20]. Here, we identified bird, canine, and equine species as hosts of this species in urban and forested areas. This species has been recorded in Brazil as showing a tendency towards choosing mammals [59,61,69], with preference for large-bodied species such as horses [5,64].

The identification of avian blood in a single specimen of *Limatus* is consistent with previous records on the genus (e.g. *L*. *durhami* and *L*. *pseudomethisticus*), which were considered as opportunistic for choosing avian and mammalian hosts, including humans [67]. The low sampling does not allow us to analyze the host preferences of this genus.

In PEP, opportunistic species (*An*. *cruzii* and *Ae*. *fluviatilis*) [61,69] or species that tend to select mammalian hosts and, occasionally, birds (*Ps*. *ferox*) [20] had their blood meals identified as the blood of humans that possibly entered the forest or lived in the vicinity of the park. The low number of positive identifications did not allow us to determine the vertebrate hosts that sustain these species, which are considered as a potential or actual vector of pathogens [11,70,71]. The contact between *An*. *cruzii* and humans and the record of non-human primates in the park [33] might indicate transmission risks of the etiological agent of simian malaria to humans [72]. Likewise, the record of bats [32] in association with the presence of *Ps*. *ferox* raises concerns about the transmission risks of the Venezuelan Equine Encephalitis Virus [11].

The knowledge of the host use patterns of Culicidae can provide answers to issues that are still poorly understood for some species (e.g. patterns of search and selection of vertebrates, their interactions with other species, and transmission risks of pathogens). Limits of temperature and storage time and better digestion conditions of blood meals investigated in the present study can contribute to the design of entomological surveillance efforts. Our results suggest that most investigated species selected hosts according to their availability in the environment, which is an important contribution given the scarcity of information on host preferences of most mosquito species, either in urban or wild habitats. Given the environmental disturbances and the possibility of emergence (or reemergence) of human pathogens, this type of effort would be instrumental to continually monitor host use of hematophagous insects.
